# Intraductal papillary carcinoma of breast with invasion: A nomogram and survival from the analysis of the SEER database

**DOI:** 10.1002/cam4.5007

**Published:** 2022-07-15

**Authors:** Chenguang Liu, Shiyang Liu, Lu Zhao, Weihong Zheng, Kun Wang, Yao Tian, Zhengwei Gui, Lin Zhang

**Affiliations:** ^1^ Department of Thyroid and Breast Surgery Tongji Hospital of Tongji Medical College of Huazhong University of Science and Technology Wuhan China

**Keywords:** intraductal papillary carcinoma, invasion, nomogram, prognosis, SEER

## Abstract

**Background:**

Intraductal papillary carcinoma (IPC) with invasion is a rare type of breast cancer. There have been few studies on its prognosis, and a nomogram that predicts the prognosis of the disease has not been described to date.

**Methods:**

Patients who were diagnosed with invasive IPC were screened from the Surveillance, Epidemiology, and End Results (SEER) database. The screened patients were randomly divided into a training cohort and a verification cohort at 7:3. A Cox proportional hazard regression model was performed to analyze the effects of different variables on the risk of death in invasive IPC. A nomogram was constructed to quantify the possibility of death. The concordance index (C‐index), calibration plots, receiver operating characteristic (ROC) curves, and decision curves analysis (DCA) were used to verify the proposed model.

**Results:**

We included a total of 803 patients diagnosed with invasive IPC, including 563 patients in the training cohort and 240 patients in the validation cohort. The median follow‐up times in the training cohort and validation cohort were 63 months (range, 2–155 months) and 61 months (range, 1–154 months), respectively. For all patients, the probability of death with invasive IPC was 1.4% within 5 years and 5.4% within 10 years. In multivariate analysis, sex, race, tumor size, lymph node status, type of treatment, and chemotherapy were related to the prognosis of invasive IPC. We constructed a nomogram to predict the possibility of death in patients with invasive IPC.

**Conclusion:**

Patients with invasive IPC had a high survival rate. The proven nomogram was helpful to both patients and clinical decision makers.

## Graphical abstract

We used the SEER database to predict the prognosis of intraductal papillary cancer of breast with invasion by constructing a nomogram

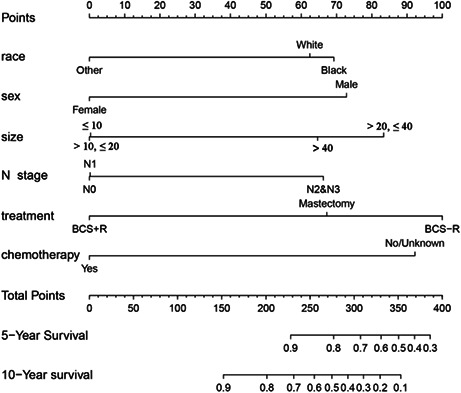

## 1 INTRODUCTION

The incidence of breast cancer has gradually increased in recent years, and it has officially replaced lung cancer as the cancer with the highest incidence in the world. Breast papillary tumors are a group of heterogeneous lesions that have some overlap in histomorphology, so there are some challenges in clinical differentiation and diagnosis. Kraus and Neubecker made a clear differentiation between benign papilloma and malignant papillary cancer for the first time.^1^ In 2019, the World Health Organization (WHO) divided papillary neoplasms into five types, including benign intraductal papilloma, papillary ductal carcinoma in situ (papillary DCIS), solid papillary carcinoma, encapsulated papillary carcinoma, and invasive papillary carcinoma. Intraductal papillary carcinoma (IPC), also referred to as noninvasive papillary carcinoma and papillary DCIS, is characterized by a complete or almost complete lack of myoepithelial cells in the papillary lobe of intraductal tumor proliferation.^2^ However, even if myoepithelial cells were widespread, the possibility of IPC could not be completely ruled out.^3^ Invasive IPC is an infrequent and special type of breast cancer and was simply considered to be invasive papillary carcinoma in previous studies.^4^ Breast invasive papillary carcinoma was defined as breast cancer with more than 90% of its invasive components having papillary structures. However, not all IPC with invasion meets the standard definition of breast invasive papillary carcinoma, and a portion of cases of IPC with invasion have invasive cancer and carcinoma in situ components.^5^ A previous article showed that IPC with invasion had a good prognosis. However, compared with other special histological types of breast cancer, such as cribriform carcinoma, mucinous carcinoma, and adenoid cystic carcinoma, the prognosis was poor.^6^

Surveillance, Epidemiology, and End Results (SEER) database is one of the most representative large tumor databases in North America.^7^, ^8^ In this study, we intended to identify clinicopathologic characteristics correlated with the incidence of death in invasive IPC using data from the SEER program. In addition, we constructed a nomogram based on the identified variables to predict the probability of death in patients with invasive IPC and verified it.

## 2 METHODS

### 2.1 Patient selection

**FIGURE 1:**
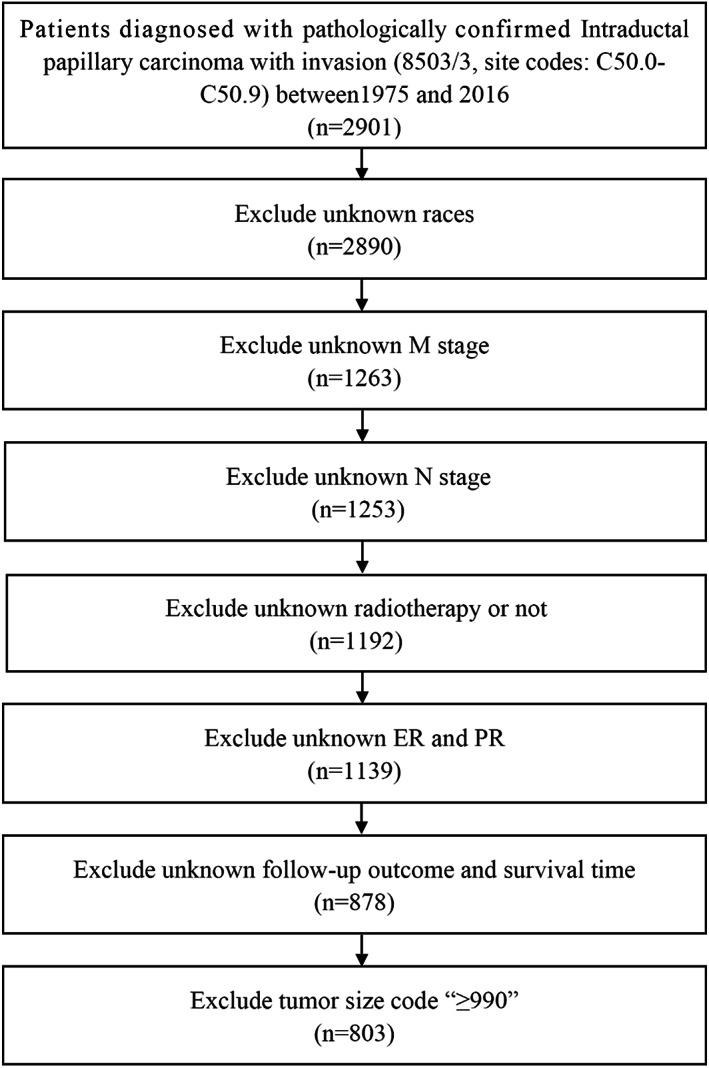
The detailed exclusion criteria and flowchart for SEER data selection

The data were obtained from the 1975–2016 database in SEER program. A total of 803 invasive IPC (site codes, C50.0–C50.9) patients were selected from the SEER*Stat program (version 8.3.4), according to the following inclusion criteria: pathologically confirmed IPC with invasion (ICD‐O‐3 8503), unilateral breast cancer, adjusted American Joint Committee on Cancer (AJCC) 6th edition N stage and M stage (diagnosis year: 2004–2015), estrogen receptor (ER), progesterone receptor (PR) status, tumor size, radiotherapy, and chemotherapy. In this analysis, patients whose survival times were unknown or without recording of adjusted AJCC 6th edition N stage and M stage were excluded. The detailed exclusion criteria and flowchart for SEER data selection are shown in Figure 1.

### 2.2 Assessment of variables

The age at the time of diagnosis was divided into three groups: younger group <45 years old, middle‐aged group between 45 and 64 years old and elderly group equal to or older than 65 years old. Tumor size was divided into four groups for analysis, including ≤10 mm, >10 and ≤20 mm, > 20 and ≤40 mm, and >40 mm. The treatment was analyzed by the combination of surgery and radiotherapy, and the three groups were mastectomy, breast‐conserving surgery combined with radiotherapy (BCS + R), and breast‐conserving surgery without radiotherapy (BCS − R).

### 2.3 Statistical analysis and method

We randomly divided all patients into a training cohort and a verification cohort. The training cohort data were used to build a predictive prognosis model, and the verification cohort data were used to internally validate the nomogram. The data of this article were analyzed by R version 3.6.2 software and SPSS 26.0. Pearson Chi‐square tests and Fisher's exact test were used to compare the classification variables between two cohorts, while independent‐samples *T*‐test was used for continuous variables. Through the analysis of univariate and multivariate Cox proportional hazard regression models, we obtained hazard ratios (HRs) and their 95% confidence intervals and analyzed the influence of variables on the risk death from invasive IPC. In order to verify the accuracy, differentiation, and clinical practicability of the nomogram, we calculated the concordance index (C‐index), drawn the receiver operating characteristic (ROC) curves, the calibration curves, and decision curves analysis (DCA). In this study, the factors with *p* < 0.1 in the univariate analysis were included in the multivariate analysis. In our study, we mainly used R packages “caret,” “rms,” “foreign,” “survival,” “timeROC,” and “dcurves.” All *p* values were two‐sided and were considered to be statistically significant when they were <0.05. We included variables with *p* < 0.05 in the nomogram.

## 3 RESULTS

### 3.1 Patient and pathological characteristics

We finally screened 803 patients with invasive IPC who were randomly divided according to a ratio of 7:3; 563 patients were included in the training set, and 240 patients were included in the verification set. Overall, most of the patients were females (96.1%) and white (68.5%). The median ages at the time of diagnosis in the training set and validation set were 65 years old (range, 24–96 years old) and 67 years old (range, 23–94 years old), respectively. For the median size of the tumor, in the training cohort, it was 16 mm (range, 1–180 mm), and in the validation cohort, it was 17 mm (range, 1–300 mm). In addition, the median time of follow‐up was 63 months (range, 2–155 months) and 61 months (range, 1–154 months), respectively. The vast majority of cases were positive for hormone receptors, accounting for 85.6% of all patients (including ER‐positive or PR‐positive). In these two cohorts, nearly half of the patients received radiotherapy (52.4% vs. 43.8%), and a small number of patients received chemotherapy (30.6% vs. 25.0%). In the training cohort, there were 87 death events (15.4%), including 12 deaths (2.1%) from invasive IPC, 54 (9.6%) deaths from other causes, and 21 (3.7%) deaths of unknown cause, and in the validation set, these data were 52 (21.7%), 4 (1.7%), 34 (14.2%), and 14 (5.8%), respectively. Generally, the characteristics of the two cohorts were similar. The demographic and clinicopathological features of the two groups of patients in this study are shown in Table 1.

**TABLE 1:** The demographic and clinicopathological characteristics of the patients with invasive IPC (including training and validation cohorts)

Clinicopathologic characteristic	Training cohort		Validation cohort		*p* value
	No.	%	No.	%	
Number	563	100	240	100	
Age at diagnosis, year					0.079
Median [Min, Max]	65 (24, 96)		67 (23, 94)		
Sex					0.058
Female	546	97.0	226	94.2	
Male	17	3.0	14	5.8	
Grade					0.614
I	156	27.7	60	25.0	
II	231	41.0	107	44.6	
III	136	24.2	51	21.3	
IV	5	0.9	2	0.8	
Unknown	35	6.2	20	8.3	
Race					0.137
Black	92	16.3	49	20.4	
White	385	68.4	165	68.8	
Other	86	15.3	26	10.8	
Tumor size, mm					0.960
Median [Min, Max]	16 (1, 180)		17 (1, 300)		
Hormone receptor subtype					0.319
ER+, PR+	422	75.0	176	73.3	
ER+, PR−	55	9.7	33	13.8	
ER−, PR+	1	0.2	0	0.0	
ER−, PR−	85	15.1	31	12.9	
Surgery					0.992
BCS	338	60.0	144	60.0	
Mastectomy	225	40.0	96	40.0	
Type of treatment					0.267
BCS + R	239	42.4	91	37.9	
BCS − R	99	17.6	53	22.1	
Mastectomy	225	40.0	96	40.0	
Size, mm					0.733
≤10	157	27.9	72	30.0	
>10, ≤20	195	34.6	75	31.2	
>20, ≤40	140	24.9	65	27.1	
>40	71	12.6	28	11.7	
Adjusted AJCC 6th N stage					0.424
N0	434	77.1	195	81.3	
N1	89	15.8	31	12.9	
N2 & N3	40	7.1	14	5.8	
Adjusted AJCC 6th M stage					0.681
M0	557	98.9	239	99.6	
M1	6	1.1	1	0.4	
Chemotherapy					0.112
None/unknown	391	69.4	180	75.0	
Yes	172	30.6	60	25.0	
Radiotherapy					0.025
None	268	47.6	135	56.2	
Yes	295	52.4	105	43.8	
Survival, month					0.594
Median [Min, Max]	63 (2, 155)		61 (1, 154)		
Death events					
Death resulting from IPC with invasion	12	2.1	4	1.7	
Death resulting from other causes	54	9.6	34	14.2	
Unknown	21	3.7	14	5.8	

Abbreviations: BCS, breast‐conserving surgery; ER, estrogen receptor; PR, progesterone receptor; R, radiotherapy.

### 3.2 Survival analysis

**FIGURE 2:**
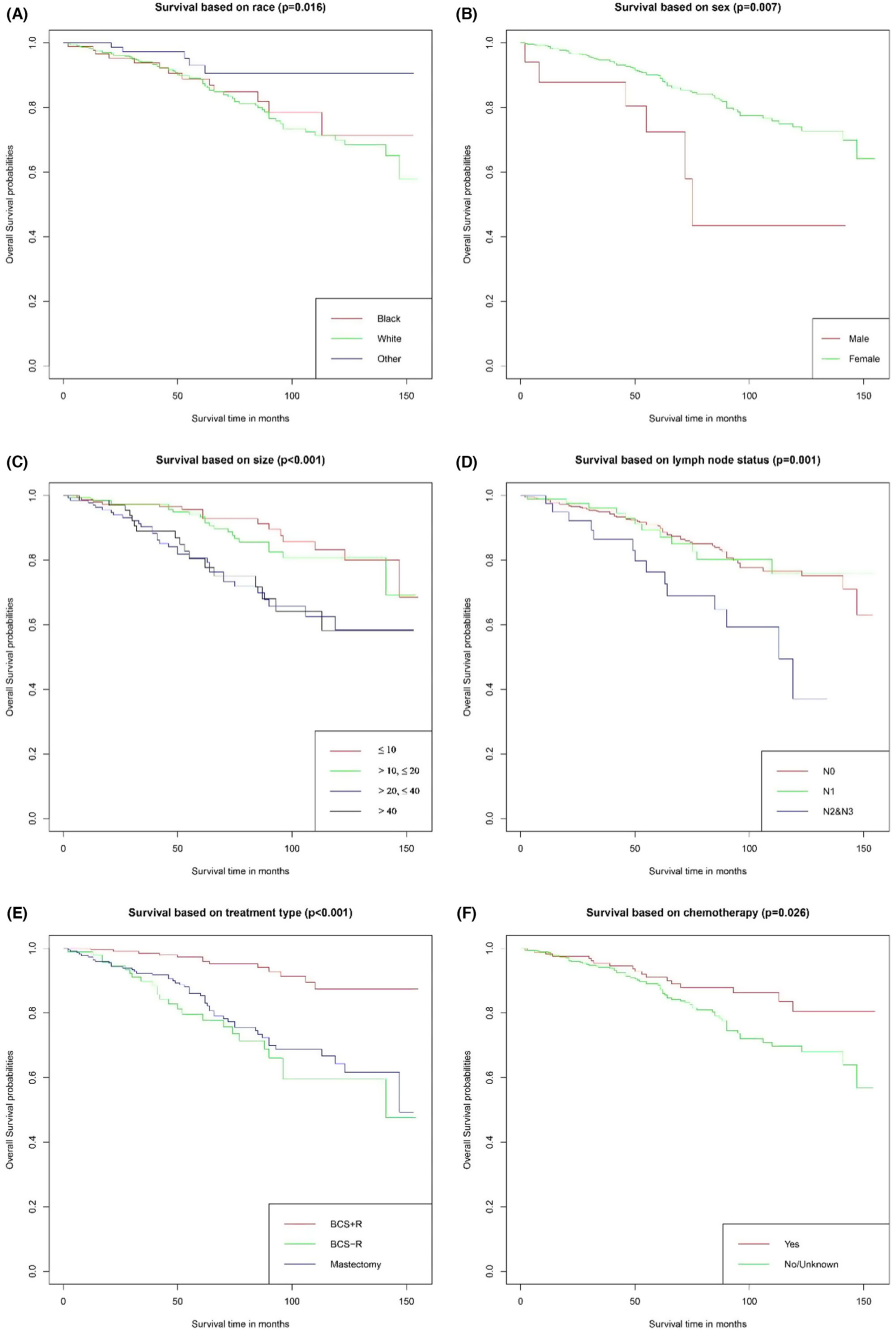
The overall survival curves of IPC with invasion in different subgroups. (A) race, *p* = 0.016; (B) sex, *p* = 0.007; (C) tumor size, *p* < 0.001; (D) lymph node status, *p* = 0.001; (E) type of treatment, *p* < 0.001; and (F) chemotherapy, *p* = 0.026.

Some of the training cohort patients with invasive IPC experienced death events after a median follow‐up of 63 months, of which five patients died of the disease itself. For all patients, the probability of death with invasive IPC was 1.4% within 5 years and 5.4% within 10 years. After univariate analysis, we screened out eight variables that might be related to prognosis (*p* < 0.1), and multivariate Cox regression analysis showed that race, sex, size of the tumor, regional lymph node status, type of treatment, and chemotherapy were significantly correlated with the risk of death (*p* < 0.05) (Table 2). We used the Kaplan–Meier method to build survival curves. Patients who received BCS + R showed better survival than those who received BCS − R or mastectomy (*p* < 0.001) (Figure 2E), and patients who received chemotherapy had a longer survival time (Figure 2F). Patients with female sex, smaller tumor size, and lower N stage also had higher survival rates (Figure 2B–D). Compared with other races, black patients had poor survival (Figure 2A).

**TABLE 2:** Univariate and Multivariate analyses of overall survival in patients with invasive IPC by COX proportional hazard regression model

Variables	Univariate analysis		Multivariate analysis	
	HR (95% CI)	*p* value	HR (95% CI)	*p* value
Age at diagnosis				
<45	Reference		Reference	
45–64	0.446 (0.176–1.134)	0.090	0.472 (0.181–1.230)	0.125
≥65	2.243 (1.030–4.885)	0.042	1.939 (0.791–4.751)	0.148
Sex				
Female	Reference		Reference	
Male	0.339 (0.148–0.778)	0.011	0.277 (0.105–0.732)	0.010
Grade				
I	Reference			
II	1.199 (0.682–2.108)	0.529		
III	1.610 (0.899–2.885)	0.109		
IV	1.015 (0.136–7.592)	0.989		
Unknown	1.278 (0.510–3.200)	0.601		
Race				
Black	Reference		Reference	
White	1.130 (0.625–2.045)	0.686	0.807 (0.425–1.535)	0.513
Other	0.392 (0.139–1.099)	0.075	0.325 (0.112–0.945)	0.039
Size, mm				
≤10	Reference		Reference	
>10, ≤20	1.259 (0.660–2.400)	0.485	1.031 (0.510–2.086)	0.933
>20, ≤40	2.959 (1.621–5.401)	<0.001	3.021 (1.552–5.881)	0.001
>40	2.925 (1.476–5.798)	0.002	2.250 (0.996–5.082)	0.051
Hormone receptor subtype				
ER+, PR+	Reference		Reference	
ER+, PR−	1.125 (0.576–2.198)	0.731	1.475 (0.720–3.023)	0.289
ER−, PR+	11.693 (1.600–85.469)	0.015	9.094 (0.923–89.634)	0.059
ER−, PR−	1.188 (0.683–2.065)	0.543	1.320 (0.711–2.450)	0.380
Type of treatment				
BSC + R	Reference		Reference	
BSC − R	5.422 (2.773–10.602)	<0.001	4.130 (2.006–8.501)	<0.001
Mastectomy	4.297 (2.331–7.921)	<0.001	2.902 (1.409–5.975)	0.004
Adjusted AJCC 6th N stage				
N0	Reference		Reference	
N1	0.972 (0.523–1.807)	0.928	1.148 (0.604–2.182)	0.674
N2 & N3	2.573 (1.438–4.606)	0.001	2.902 (1.409–5.975)	0.004
Adjusted AJCC 6th M stage				
M0	Reference			
M1	1.058 (0.147–7.603)	0.956		
Chemotherapy				
Yes	Reference		Reference	
No/unknown	1.789 (1.065–3.008)	0.028	2.659 (1.326–5.333)	0.006

### 3.3 Nomogram

**FIGURE 3:**
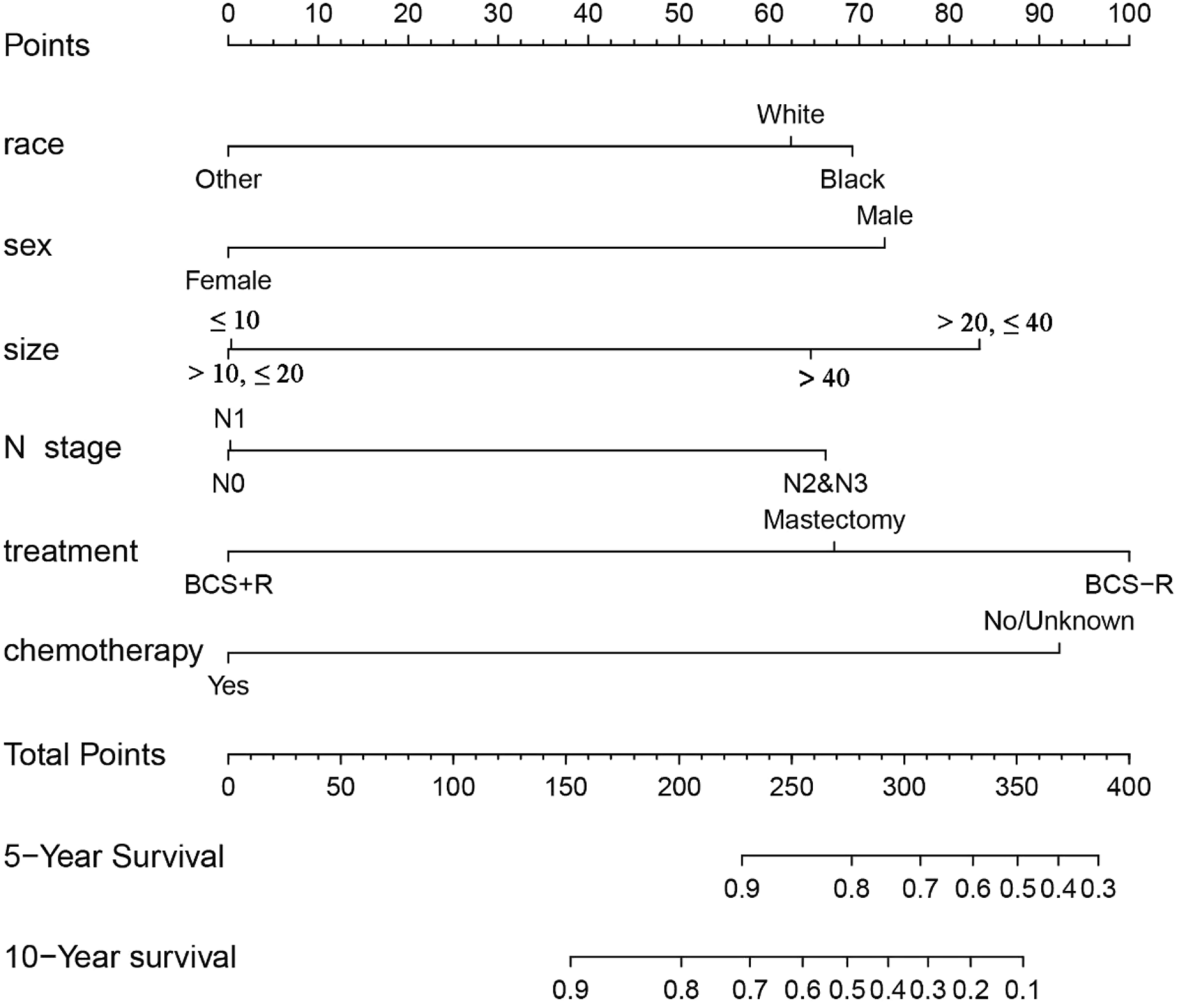
Prognostic nomogram for predicting the 5‐ and 10‐year probability of death resulting from IPC with invasion.

We used a Cox regression model to determine the factors that affect the overall survival (OS) of patients with invasive IPC to construct a nomogram that can predict its prognosis. Sex, race, tumor size, chemotherapy, type of treatment, and adjusted AJCC 6th edition N stage were included in the nomogram. The nomogram is displayed in Figure 3. When we used the nomogram, we first needed to determine its position on different variable axes, find the corresponding points on the top axis, add the point values of all variables together, and draw a vertical line down based on this sum point to predict the risk of 5‐year or 10‐year death rate in patients with invasive IPC.

### 3.4 Internal validation

**FIGURE 4:**
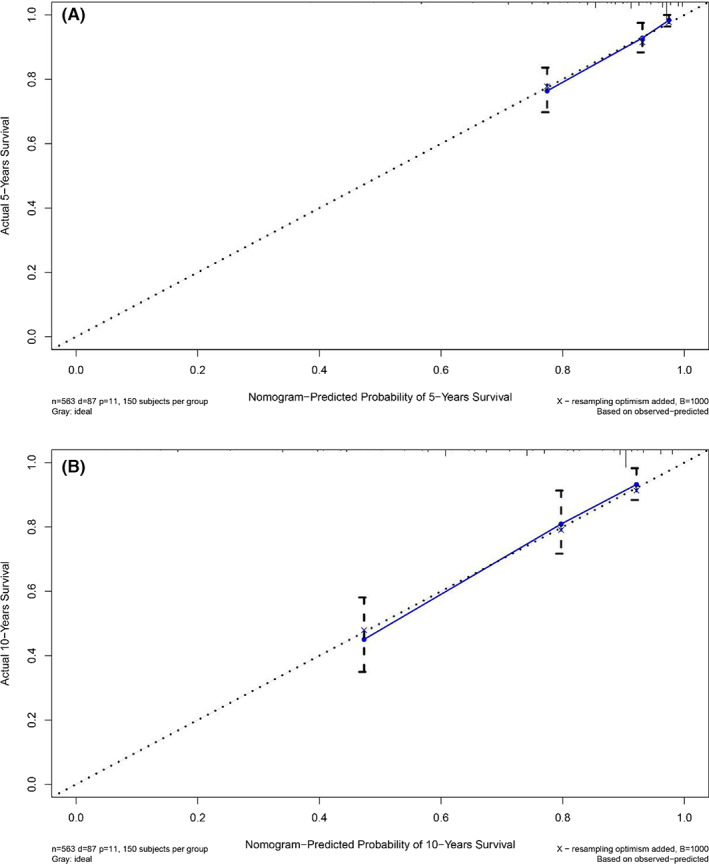
Calibration plots of OS‐associated nomograms in training cohort. (A) The calibration plots in 5‐year OS; (B) The calibration plots in 10‐year OS.

**FIGURE 5:**
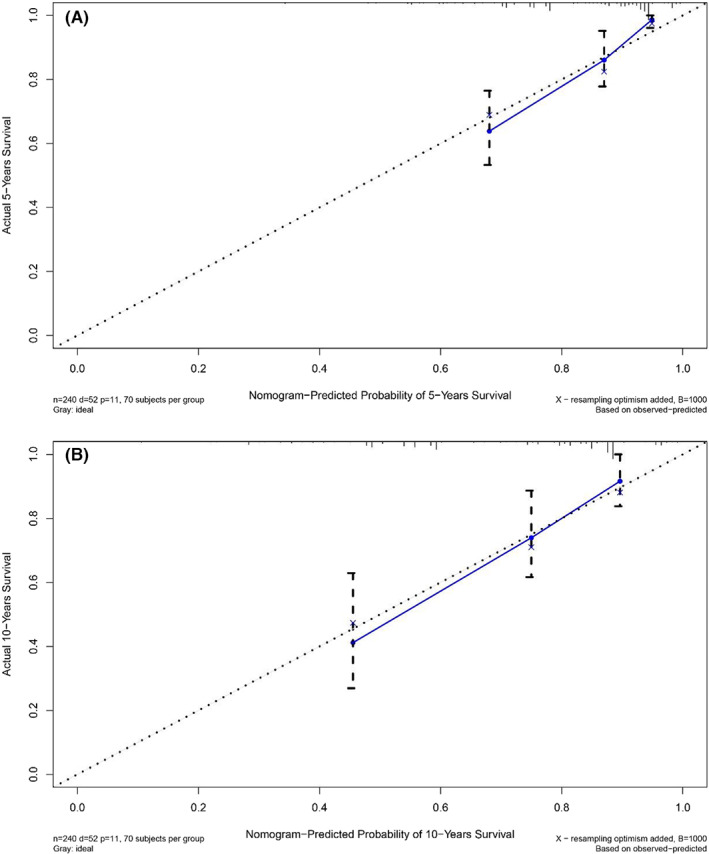
Calibration plots of OS‐associated nomograms in validation cohort. (A) The calibration plots in 5‐year OS; (B) The calibration plots in 10‐year OS.

When the C‐index >0.7, it was considered that the prediction model had better accuracy.^9^, ^10^ The C‐index of the nomogram in the training cohort was 0.768 (95%CI = 0.715–0.821) and that in the validation cohort was 0.761 (95%CI = 0.702–0.820). The 45‐degree slash in the calibration chart was regarded as a more perfect result. Whether in the training cohort or in the verification cohort, the calibration charts showed that there was good agreement between the 5‐year and 10‐year survival prediction probability and the actual probability (Figures 4A,B and 5A,B).

**FIGURE 6:**
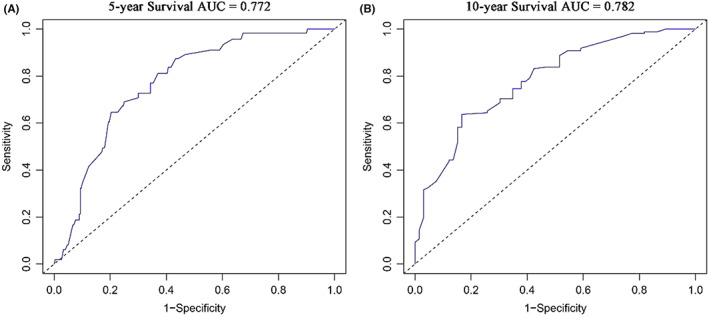
ROC curves for evaluating the nomogram in the training cohort. (A) ROC curve of the 5‐year survival nomogram; (B) ROC curve of the 10‐year survival nomogram. AUC, area under ROC curve; ROC, receiver operating characteristic.

**FIGURE 7:**
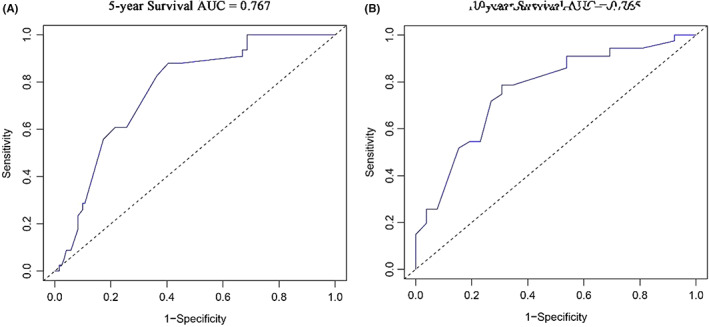
ROC curves for evaluating the nomogram in the validation cohort. (A) ROC curve of the 5‐year survival nomogram; (B) ROC curve of the 10‐year survival nomogram. AUC, area under ROC curve; ROC, receiver operating characteristic.

In addition, we also evaluated the specificity and sensitivity of the nomogram by drawing ROC curves. The area under the ROC curves (AUC) in the training cohort showed good accuracy in predicting the risk of death at 5 and 10 years (5‐year: 0.772, 95%CI = 0.709–0.835; 10‐year: 0.782, 95%CI = 0.706–0.858) (Figure 6A,B), and the results were similar in the validation cohort (5‐year: 0.767, 95%CI = 0.687–0.848; 10‐year: 0.765, 95%CI = 0.647–0.883) (Figure 7A,B).

**FIGURE 8:**
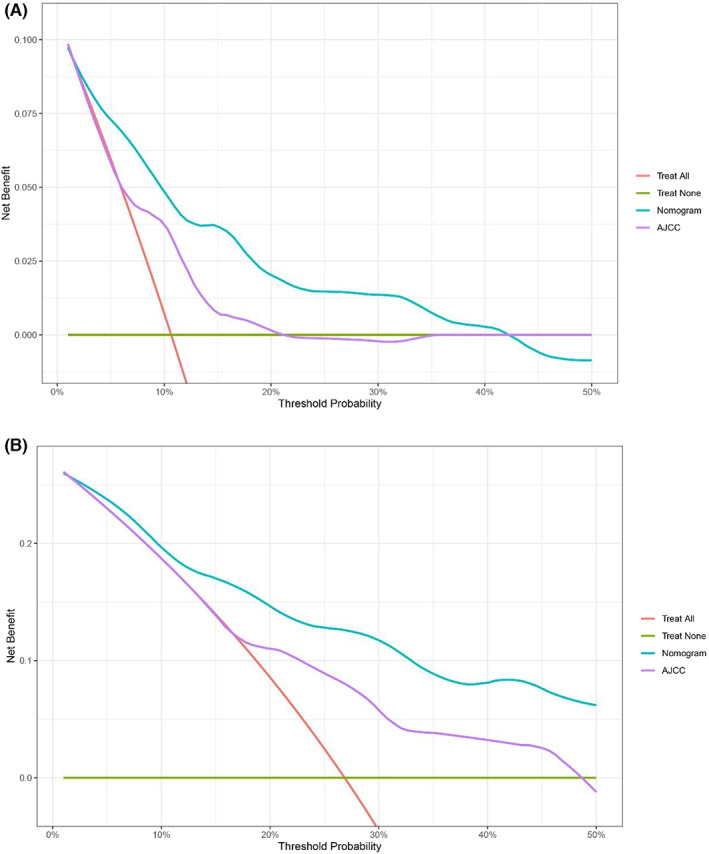
The decision curves analysis for assessing clinical utility of the nomogram in predicting invasive IPC 5‐year OS (A) and 10‐year OS (B) in training cohort.

**FIGURE 9:**
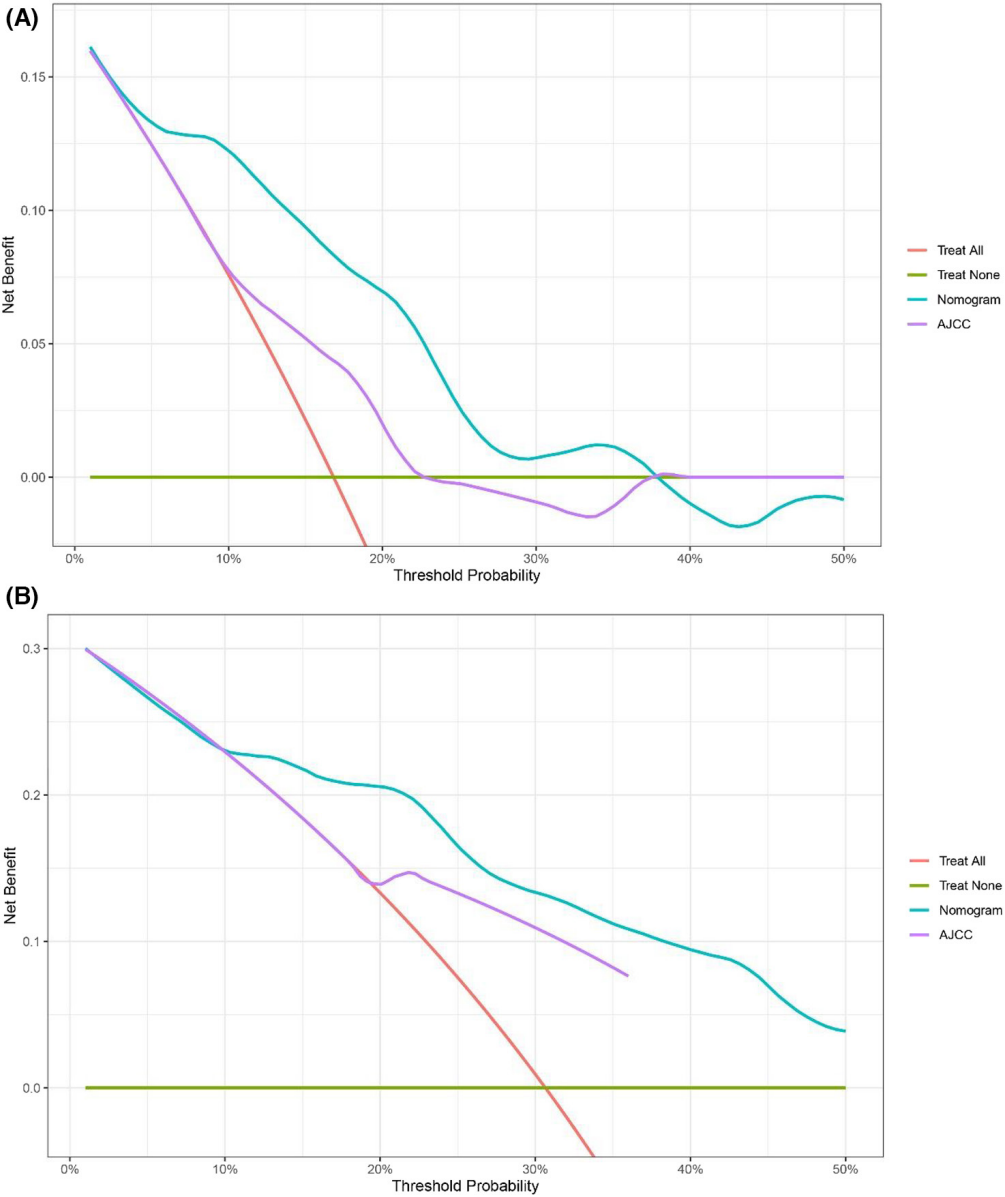
The decision curves analysis for assessing clinical utility of the nomogram in predicting invasive IPC 5‐year OS (A) and 10‐year OS (B) in validation cohort.

Additionally, in order to further evaluate the clinical practicability of the nomogram, we drew DCA curves of the two cohorts and compared the nomogram with the AJCC staging system. When the line segment represented by the nomogram was outside the two extreme curves (red and green segment in Figures 8, 9), it showed that it had application value, and the farther away it was, the higher the application value was. The DCA curves showed that the nomogram could better predict the 5‐year and 10‐year OS of IPC with invasion than the AJCC staging system (Figures 8A,B and 9A,B).

## 4 DISCUSSION

Invasive IPC is a rare breast neoplasm. Breast papillary carcinoma accounts for 0.5%–1% of breast cancers.^11^ Without invasion, the ratio of papillary DCIS to papillary lesions was approximately 10%.^12^ It had been reported that invasive papillary carcinoma was rarer.^13^ In terms of histological morphology, IPC with invasion was characterized by a dendritic fibrous vascular core supporting epithelial proliferation, lined with layered columnar tumor epithelial cells, and no complete myoepithelial layer in the papilla or epithelial hyperplasia.^2^ For a long time, because of the confused histological classification and scarcity, few researchers have focused on the prognosis of invasive IPC. We used a large sample of cases in the SEER database to analyze the effect of clinical and pathological characteristics on the prognosis of patients with invasive IPC and to analyze the benefits of different treatments.

In the multivariate analysis, sex, race, tumor size, chemotherapy, type of treatment, and adjusted AJCC 6th edition N stage were significantly correlated with OS. Some studies have shown that recurrence and metastasis of disease are associated with a higher histological grade.^14^ However, we did not find that histological grade was a factor affecting the prognosis of patients. In our study, we found that the greater the tumor load was, the worse the prognosis of invasive IPC, which was basically consistent with previous studies. In addition, distant metastasis was less likely to occur. Researchers have found that IPC with invasion rarely involves lymph nodes, its overall prognosis is good, and they believe that routine evaluation of axillary sentinel lymph nodes is not recommended in patients with IPC receiving BCS.^15^ However, in our study, patients had a relatively high ratio of lymph node metastasis despite invasion (21.6%), suggesting that approximately 20% of cases of IPC with invasion might be ignored or even delayed the disease if we gave up the assessment of sentinel lymph nodes. Therefore, we believe that, in the course of clinical work, axillary sentinel lymph node biopsy in these patients is still necessary.

As a special subtype of breast cancer, invasive IPC commonly shows the absence of myoepithelial markers in immunohistochemical analysis,^16^ which indicates that it is partly consistent with the characteristics of invasive carcinoma. Therefore, in the immunohistochemistry of IPC, we paid more attention to muscle epithelial markers, such as P63, CK14, and CK5/6.^17^, ^18^ In 2015, a study found that there was a visible difference in the existence of the mitotic marker cyclin B1 between benign and malignant papillary breast lesions. However, because of the low natural level of expression and heterogeneity of this marker, it is difficult for it to play a substantial role in conventional clinical practice.^19^ In addition, some studies have shown that ER and PR are typically expressed in IPC,^2^, ^17^ whether it is accompanied by invasion. A high‐resolution microarray‐based comparative genomic hybridization study showed that breast papillary carcinomas presented fewer genomic aberrations than invasive breast cancer of nonspecial type matched for both grade and ER. In addition, the genomic maps of the three subtypes of encapsulated papillary carcinoma, solid papillary carcinoma, and invasive intraductal papillary carcinoma were very similar. Therefore, from a genomic point of view, papillary carcinoma is a homogenous and particular histological type of breast cancer.^20^ In our study, most of the cases expressed both ER and PR (74.5%), which were roughly the same as the results of previous studies.^17^ In multivariate Cox regression analysis, hormone receptor subtypes had no significant effect on OS. From this point of view, it might be difficult to distinguish invasive IPC from simple IPC in terms of clinical features.

Regarding the type of diagnosis and treatment, there have been many disputes in the past. The results of core needle biopsy (CAB) could not clearly distinguish benign and malignant papillary tumors. The First and Second International Consensus Conference on lesions of uncertain malignant potential in the breast (B3 lesions) showed that the consensus recommendations of experts on papillary lesions were basically the same; that is, breast papillary lesions that could be seen on imaging should be removed with vacuum‐assisted biopsy (VAB).^21^, ^22^ Papillary tumors diagnosed by CAB were more than 30% likely to escalate to malignancy when they were accompanied with atypical lobular hyperplasia (ALH) or atypical duct hyperplasia (ADH), and surgical resection was recommended.^23^, ^24^ Our study showed that the prognosis of patients with chemotherapy was significantly better than that of patients without chemotherapy, which suggested that patients with invasion should not be too optimistic in terms of treatment. It is well known that eligible breast cancer patients who receive BCS + R have similar survival rates to those who undergo mastectomy.^25^ Therefore, early patients were more likely to choose BCS + R. The current research is more focused on improving the accuracy of radiotherapy without reducing the survival of patients.^26^, ^27^ In our study, we found that BCS + R was strongly correlated with good prognosis compared with mastectomy with or without radiation. Interestingly, BCS + R was even found to improve OS in cancer in situ,^28^ which was similar to our conclusion. This finding indicated that, in clinical decisions, BCS + R might be a good surgical method for IPC

The nomogram visualized the clinical indices of different patients after complex calculation so that it could predict the prognosis of patients intuitively and easily. The model is mature and has been used in many diseases, such as colorectal cancer,^29^ intrahepatic cholangiocarcinoma,^30^ nasopharyngeal carcinoma,^31^ and early mucinous breast cancer.^32^ For all patients, we found that the 5‐year and 10‐year rates of death resulting from invasive IPC were 1.4% and 5.4%, respectively. The lower mortality rate indicated that even IPC with invasion had a good prognosis. However, doctors cannot accurately predict the prognosis of patients. Therefore, the establishment of the nomogram was necessary and would be helpful for the clinical decision‐making process. We constructed a predictive model to integrate the clinicopathological indicators related to the prognosis in Figure 3. This could help clinicians to personally evaluate the prognosis of IPC patients with invasion. Internal validation showed that there was a high consistency between prediction and reality.

Objectively speaking, our study had some limitations. The SEER database could not screen patients with neoadjuvant chemotherapy, and there was no way to know the target area, dose of radiotherapy, or specific size of the infiltration component. Although SEER could not provide all the prognostic information, it is still a widely used database for the construction of nomograms.

## 5 CONCLUSION

IPC with invasion is a rare breast disease. In the case of invasion, the prognosis of IPC is still good, there is less lymph node metastasis, and local recurrence may occur. The treatment of BCS + R may be more suitable for invasive IPC. The nomogram model provides an intuitive and applicable tool for the prognosis of IPC with invasion.

## AUTHOR CONTRIBUTIONS

Lin Zhang: Conceptualization, Methodology. Chenguang Liu: Data curation, Statistical analysis, Software, Writing—Original draft preparation. Shiyang Liu: Visualization, Investigation, Statistical analysis, Writing—Reviewing and Editing. Lu Zhao: Supervision, Resources. Weihong Zheng: Visualization, Software. Kun Wang: Writing—Reviewing and Editing. Yao Tian: Software, Validation. Zhengwei Gui: Investigation.

## CONFLICT OF INTEREST

The authors declare that we have no conflict of interests.

## ETHICAL STATEMENT

The data of this study came from a public database and did not involve any ethical issues.

## Data Availability

The data of this study can be obtained from Surveillance, Epidemiology, and End Results, https://seer.cancer.gov/
